# Smoking in Top-Grossing US Movies, 2011

**DOI:** 10.5888/pcd9.120170

**Published:** 2012-09-27

**Authors:** Stanton A. Glantz, Anne Iaccopucci, Kori Titus, Jonathan R. Polansky

**Affiliations:** Author Affiliations: Anne Iaccopucci, Kori Titus, Breathe California of Sacramento-Emigrant Trails, Sacramento, California; Jonathan R. Polansky, Onbeyond LLC, Fairfax, California.

## Abstract

We reviewed the number of incidents of tobacco use (almost exclusively smoking) depicted in movies in the United States in 2011 to compare that with previously reported trends. We counted use or implied use of a tobacco product by an actor in all movies whose box office gross ranked in the top 10 for at least 1 week. Total tobacco incidents per movie rose 7% from 2010 to 2011, ending 5 years of decline; incidents rose 34% per movie rated G, PG, or PG-13 and 7% per R-rated movie. The reversal of progress toward less onscreen smoking in youth-rated movies underscores the need to rate movies with tobacco imagery as R, establishing an industry-wide market incentive to keep youth-marketed movies tobacco-free.

## Objective

Exposure to onscreen smoking causes youth smoking initiation (1). The Department of Health and Human Services’ strategic plan includes the goal of reducing youth exposure to onscreen smoking (2). Although depictions of tobacco use in movies declined between 2005 and 2010, and 3 of the 6 Motion Picture Association of America (MPAA) member companies published policies designed to discourage tobacco use in their movies, movies continue to deliver billions of smoking images to adolescents (3). We report the number of incidents of tobacco use in movies released in 2011 and how 2011 relates to previously reported (3) long-term trends.

## Methods

To monitor tobacco appearances in movies, Thumbs Up! Thumbs Down! (TUTD), a project of Breathe California of Sacramento-Emigrant Trails, counts occurrences of tobacco “incidents” in US top-grossing movies each year. TUTD uses trained monitors to count tobacco incidents in all movies that are among the 10 top-grossing movies in any calendar week (83% of all movies exhibited in the United States and 98% of tickets sold in 2002–2008). An incident is 1 use or implied use of a tobacco product (almost exclusively smoking) by an actor. We calculated impressions (1 person seeing 1 tobacco use incident 1 time) for each movie by multiplying tickets sold for the movie by the number of incidents. Tickets sold were calculated by dividing the domestic box office gross receipts reported for the movie (www.boxofficemojo.com) by the average US ticket price (www.natoonline.org) in the year the movie was released. We compared results in 2011 with 2010 and with long-term trends (4).

## Results

**Figure 1:**
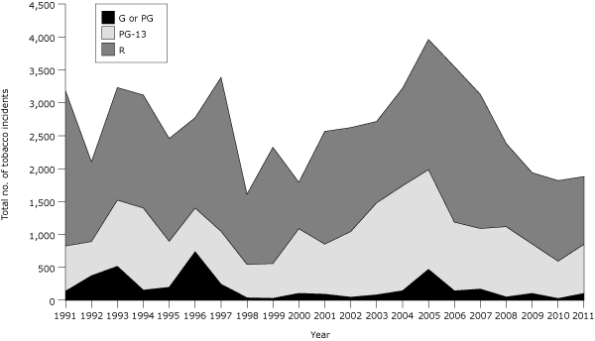
Tobacco incidents in top-grossing US movies by year and movie rating, 1991–2011. Top-grossing movies were those that were among the 10 top-grossing movies in any calendar week of the year. An incident of tobacco use is 1 use or implied use of a tobacco product (almost exclusively smoking) by an actor. Historical data are from our earlier report (3)

In 2011, 134 movies were among the 10 top-grossing movies for at least 1 week. The total number of tobacco incidents rose 3% (from 1,819 to 1,881) from 2010 to 2011 despite there being 5 fewer movies in the 2011 sample than the 139 in 2010 (Figure 1). Overall, the number of tobacco incidents per movie increased 7% (from 13.1 to 14.0). Changes varied by MPAA rating. Incidents per G and PG movie climbed 311% (from 0.8 to 3.2) and per PG-13 movie, 9% (from 10.7 to 11.6); tobacco incidents per youth-rated movie (G, PG, and PG-13 combined) rose 34% (from 6.5 to 8.8). Incidents per R-rated movie increased 7% (26.0 to 27.8).

**Table:** 

Year	Movie Rating			Total
	G or PG	PG-13	R	
**1991**	140	686	2,357	3,183
**1992**	375	518	1,212	2,105
**1993**	518	1,004	1,709	3,231
**1994**	157	1,245	1,717	3,119
**1995**	200	697	1,561	2,458
**1996**	740	662	1,372	2,774
**1997**	246	802	2,338	3,386
**1998**	41	505	1,067	1,613
**1999**	32	527	1,766	2,325
**2000**	108	980	705	1,793
**2001**	95	759	1,712	2,566
**2002**	50	995	1,579	2,624
**2003**	85	1,395	1,237	2,717
**2004**	147	1,593	1,480	3,220
**2005**	472	1,513	1,977	3,962
**2006**	146	1,041	2,362	3,549
**2007**	173	918	2,038	3,129
**2008**	53	1,066	1,263	2,382
**2009**	107	749	1,083	1,939
**2010**	30	565	1,224	1,819
**2011**	107	745	1,029	1,881

Ending a multiyear upward trend (3), no substantial change occurred in the share of movies that were free of tobacco depictions in 2011 compared with 2010. Of top-grossing movies, 54% (72) were free of tobacco depictions in 2011 compared with 56% (78) in 2010. Among movies rated G or PG, 82% (27 of 33) were smoke-free in 2011 compared with 89% (34 of 38) in 2010. Among PG-13 movies, 53% (34 of 64) were smoke-free in 2011 compared with 57% (30 of 53) in 2010. Among R movies, 30% were smoke-free in both 2011 (11 of 37) and 2010 (14 of 47).

**Figure 2:**
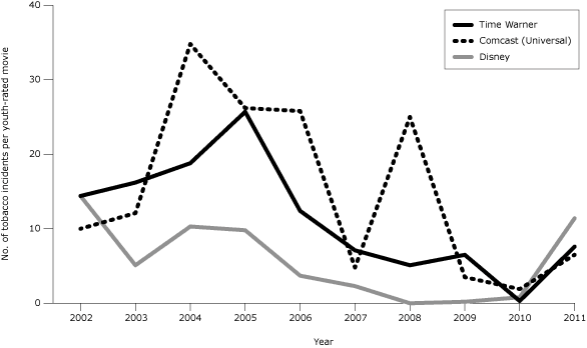
Tobacco incidents per youth-rated top-grossing US movie among companies with published policies related to tobacco in youth-rated movies, 2002–2011. Time Warner adopted its policy in 2005, Comcast in 2007, and Disney in 2004. Youth-rated movies are those rated G, PG, or PG-13 by the Motion Picture Association of America. Top-grossing movies were those that were among the 10 top-grossing movies in any calendar week of the year. An incident is 1 use or implied use of a tobacco product (almost exclusively smoking) by an actor. Historical data are from our earlier report (3).

**Figure 3:**
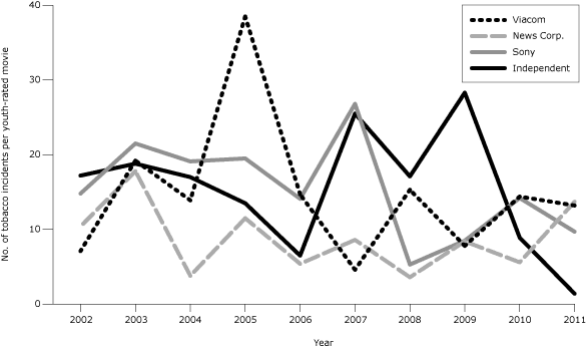
Tobacco incidents per youth-rated top-grossing US movie among companies without published policies related to tobacco in youth-rated movies, 2002–2011. Youth-rated movies are those rated G, PG, or PG-13 by the Motion Picture Association of America (MPAA). Top-grossing movies were those that were among the 10 top-grossing movies in any calendar week of the year. An incident is 1 use or implied use of a tobacco product (almost exclusively smoking) by an actor. Independent companies are those that are not members of the MPAA. Historical data are from our earlier report (3).

From 2005 through 2010, the 3 MPAA-member companies that had publicly available policies designed to discourage (but not eliminate) smoking in their movies (Comcast [Universal] [5], Disney [6], and Time Warner [7]) reduced tobacco incidents per youth-rated movie by more than 90%, to an average of 1 incident per movie (Figure 2). MPAA-member companies without such policies averaged reductions less than half as large (Figure 3), to an average of 11 incidents per movie (3). Companies with policies on average had 7.6 more tobacco incidents per youth-rated movie in 2011 than in 2010, to average 8.5 incidents per movie in 2011, while companies without policies had 1.3 fewer incidents, to average 11.9 incidents per movie in 2011. From 2010 to 2011, across companies with policies, the percentage of youth-rated movies that were tobacco-free declined by 17 percentage points (from 89% to 72%). Companies without policies also showed a retreat (from 64% to 55%). As of June 2012, no company had a blanket policy against including smoking or other tobacco imagery in its movies.

**Table:** 

Company	2002	2003	2004	2005	2006	2007	2008	2009	2010	2011
Comcast (Universal)	10.0	12.1	34.8	26.2	25.8	4.8	25.0	3.5	1.9	6.5
Disney	14.4	5.1	10.3	9.8	3.7	2.3	0.0	0.2	0.8	11.4
Time Warner	14.4	16.2	18.8	25.7	12.4	7.1	5.1	6.5	0.3	7.6

**Table:** 

Company	2002	2003	2004	2005	2006	2007	2008	2009	2010	2011
News Corp.	10.4	17.8	3.8	11.5	5.4	8.6	3.6	8.4	5.6	13.7
Sony	14.8	21.5	19.1	19.5	14.1	26.8	5.3	8.5	14.2	9.7
Viacom	7.1	19.2	13.9	38.5	14.7	4.6	15.3	7.8	14.4	13.2
Independent	17.2	18.8	17.0	13.5	6.5	25.5	17.1	28.3	8.9	1.4

In 2011, youth-rated movies delivered 10.7 billion tobacco impressions in theatrical release, double that in 2010 (5.5 billion). Youth-rated movies in 2011 delivered 68% of all in-theater tobacco impressions (10.7 billion out of 15.9 billion) compared with 39% (5.5 billion out of 14.2 billion) in such movies in 2010.

Tobacco brands continued to appear in top-grossing movies in 2011: Marlboro, Copenhagen, Camel, and Kool were used by the lead actors and a supporting actor in 5 top-grossing movies (4 R-rated, 1 PG-13–rated), all distributed by Sony. In 2010, Marlboro, Camel, Winston, and Newport appeared on billboards or packaging in 4 top-grossing movies (3 R-rated, 1 PG-rated) distributed by Disney, Liberty Media, Lionsgate, and Sony.

## Discussion

The reversal in the previous multiyear downward trend in onscreen tobacco use that occurred from 2005 to 2010 (3) means that movies in 2011 contributed more to promoting youth smoking than in previous years and that the motion picture industry is no longer progressing toward the goal of reducing onscreen depictions of tobacco use (2). Thirty-six states offer movie producers hundreds of millions of dollars in subsidies covering about 25% of production costs (8). About two-thirds of subsidies for top-grossing movies go to productions with smoking; one-third support youth-rated movies with smoking. State and local health departments should work with policy makers to harmonize movie subsidy programs with the state’s interest in reducing rates of tobacco use among youth by limiting eligibility for subsidies to tobacco-free productions.

The growth in onscreen tobacco use in 2011 reversed years of progress toward tobacco-free youth-rated movies, particularly among the 3 studios with policies meant to discourage onscreen tobacco imagery. This development reinforces the need to modernize the MPAA rating system to give movies with any tobacco use an R rating to create a sustained, industry-wide market incentive to keep movies that are marketed to youth tobacco-free (1). There should be exceptions when the presentation of tobacco clearly and unambiguously reflects the dangers and consequences of tobacco use or is necessary to represent the smoking of a real historical figure. Youth see some R-rated movies; therefore, removing tobacco imagery from new youth-rated movies will greatly reduce, but not eliminate, youth exposure to onscreen smoking and other tobacco use. We recommend that an antitobacco message run before any movie with tobacco imagery, in all channels (eg, theatrical exhibition, broadcast, pay-per-view, DVD, Blu-ray, Internet stream and download). We also recommend that moviemakers adopt complementary policies to certify that they received no payoffs for depicting tobacco use and to end depiction of tobacco brands.
